# Alcohol Abuse as a Silent Risk Factor for Purpura Fulminans and the Importance of Pneumococcal Vaccination

**DOI:** 10.7759/cureus.39522

**Published:** 2023-05-26

**Authors:** Jamal C Perry, Rachel L Pindek, Avtar Sur, Nabin KC, John Zeibeq

**Affiliations:** 1 Medicine, Brookdale University Hospital Medical Center, Brooklyn, USA; 2 Osteopathic Medicine, New York Institute of Technology College of Osteopathic Medicine, Old Westbury, USA; 3 Internal Medicine, Brookdale University Hospital Medical Center, Brooklyn, USA; 4 Critical Care Medicine, One Brooklyn Health System-Interfaith Medical Center, New York, USA; 5 Pulmonary and Critical Care Medicine, Brookdale University Hospital Medical Center, Brooklyn, USA

**Keywords:** vaccination, disseminated intravascular coagulopathy, purpura fulminans, streptococcus pneumoniae, alcohol abuse

## Abstract

Purpura fulminans (PF) is a rare presentation of disseminated intravascular coagulopathy (DIC) and a life-threatening complication of septic shock. DIC can present with bleeding and thrombosis in acute settings, making its management exceptionally challenging. Common causative organisms include *Neisseria meningitidis*,* Streptococcus pneumoniae*, and*Haemophilus influenzae*.

We report a case of a 47-year-old patient with a history of alcohol abuse and marijuana use with a bizarre presentation of copious diarrhea and altered mental status. The patient was subsequently admitted to the intensive care unit (ICU) for acute respiratory failure and septic shock secondary to *Streptococcus pneumoniae* bacteremia complicated by DIC. Unfortunately, the patient's condition worsened with multiorgan failure and purpura fulminans, leading to extensive necrosis of all his extremities, with the involvement of his lips, nose, and genitals. Unfortunately, despite aggressive interventions, he continued to deteriorate and ultimately was transitioned to comfort care before he expired.

In the literature, there is only one reported case of PF in an individual with alcohol abuse. However, the frequency and severity of pneumococcal infections in individuals with a history of alcohol abuse are much higher than in the general population. PF is a devastating complication of *Streptococcus pneumoniae, *with a mortality of 43%. We hope that this case will continue highlighting the importance of vaccinating patients with a history of alcohol abuse with the pneumococcal vaccine.

## Introduction

Alcohol abuse is a significant public health issue, with numerous consequences for physical and mental health. Alcohol abuse disorder is a well-known risk factor for community-acquired pneumonia associated with poor outcomes and high mortality rates. These individuals are at greater risk of infection with *Streptococcus pneumoniae* [1]. One of the lesser-known associations of alcohol abuse is purpura fulminans (PF).

PF is a life-threatening presentation of disseminated intravascular coagulation (DIC) involving dermal vascular thrombosis and vascular collapse associated with hemorrhagic tissue necrosis [2]. It can cause small-to-medium-sized blood vessel thrombosis, and in severe cases, it may culminate into extensive large-vessel thrombosis [2,3]. *Streptococcus pneumoniae* is commonly associated with PF [3]. In addition, PF is associated with high mortality with multiorgan failure, and symmetric peripheral gangrenous limbs may ultimately require amputation [4,5].

In this case report, we describe a male patient with a history of chronic alcohol abuse who presented with DIC secondary to septic shock and severe PF after a few days in the intensive care unit (ICU), highlighting the potential link between alcohol abuse and the development of this condition. Additionally, it emphasizes the importance of vaccination as a preventive measure against infections that can trigger purpura fulminans, as our patient had not received appropriate vaccinations.

## Case presentation

A 47-year-old previously healthy male with a history of moderate alcohol abuse presented to the emergency department with a one-day history of altered mental status, shortness of breath, and copious yellow watery diarrhea. On physical examination, the patient had a fever of 102.8°F, a pulse of 135 per minute, blood pressure of 152/99, and a respiratory rate of 26 per minute. The patient was intubated for acute respiratory failure and septic shock and transferred to the ICU.

**Figure 1:**
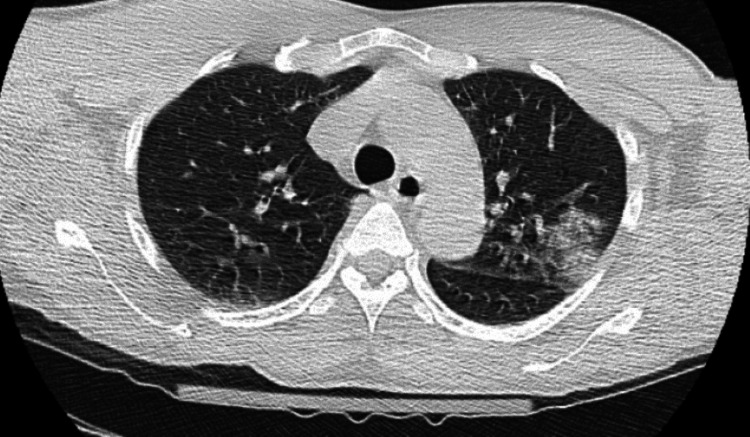
CT chest demonstrating left upper lobe pneumonia

CT head without contrast on admission was negative, and CT chest without contrast showed left upper lobe pneumonia (Figure 1). CT abdomen showed perinephric stranding.

Despite adequate fluid resuscitation with a 5 L bolus in the first several hours in the emergency department, he required IV pressors with norepinephrine, vasopressin, phenylephrine, and angiotensin. Initially, the patient was started on cefepime and vancomycin and later switched to ceftriaxone 2 g every 12 hours and metronidazole 500 mg every 12 hours, and vancomycin 1 g daily was continued.

**Figure 2:**
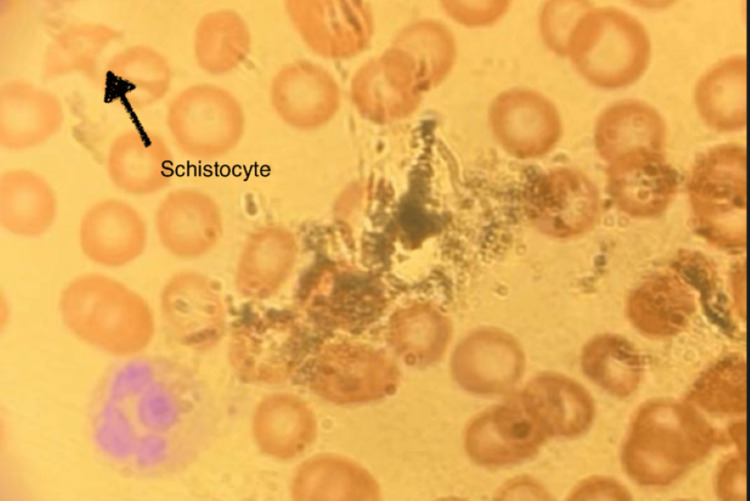
Peripheral blood smear of the patient

Clinical presentation and laboratory results (grossly elevated partial thromboplastin time {PTT}, international normalized ratio {INR}, and prothrombin time {PT} and low platelet and fibrinogen levels) suggested DIC (Table 1). As a result, a peripheral blood smear was reviewed, which was notable for polychromasia, burr cells, occasional schistocytes, several bands with toxic granulation, and decreased platelets of <2 per high-power field, with no platelet clumping (Figure 2). His hemoglobin dropped from 12 to 7.7. He received 10 units of cryoprecipitate, one unit of platelets, two units of fresh frozen plasma (FFP), and two units of RBC. The patient's coagulation cascade improved temporarily.

**Table 1:** Relative laboratory results corresponding to the patient's presentations WBC, white blood cell; BUN, blood urea nitrogen; INR, international normalized ratio; PTT, partial thromboplastin time; PT, prothrombin time; ALP, alkaline phosphatase; AST, aspartate aminotransferase; ALT, alanine aminotransferase; CRP, C-reactive protein; C-ANCA, cytoplasmic antineutrophil cytoplasmic antibodies; P-ANCA, perinuclear antineutrophil cytoplasmic antibody

Laboratory Data	Values	Reference Range
WBC	19.0×10^3^/uL (H)	4.1-10.1×10^3^/uL
Neutrophils	95.80%	44.5%-73.4%
Lymphocytes	3.80%	17.8%-42.0%
Monocytes	0.60%	5.7%-11.2%
Eosinophils	0.10%	0.2%-6.0%
Basophils	0.20%	0.3%-1.2%
Neutrophils absolute	18.20×10^3^/uL	1.40-6.80×10^3^/uL
Hemoglobin	11.1 g/dL	12.9-16.7 g/dL
Hematocrit	34.9% (L)	40%-47%
CRP	15.50 mg/dL	0.5-1.00 mg/dL
Troponin	45 ng/ml	0.034-0.12 ng/ml
D-dimer	69,000 ng/ml	≤230 ng/ml
C-ANCA	<1:20 (negative)	<1.20
P-ANCA	<1:20 (negative)	<1.20
Creatinine	5.5 mg/dL (HH)	0.66-1.25 mg/dL
BUN	37 mg/dL (H)	9.0-20.0 mg/dL
Sodium	134 mmol/L	133-145 mmol/L
ALT	180 U/L	<50 U/L
AST	328 U/L	17-59 U/L
ALP	175 U/L	38.0-126. U/L
Bilirubin total	1.1 mg/dL	0.2-1.3 mg/dL
Platelets	30×10^3^/uL	153-328×10^3^/uL
PTT	200 seconds	23.5-35.5 seconds
PT	47 seconds	9.2-12.8 seconds
INR	4	0.70-1.20
Complement 3	51 mg/dL	82-167 mg/dL
Complement 4	16 mg/dL	12-38 mg/dL
Lactate	7.20 mmol/L	0.70-2.10 mmol/L

Urine analysis was significant for hematuria and proteinuria. Autoimmune causes, including Sjögren's syndrome, Smith antibodies, anti-scleroderma 70, anti-DNA, ribonucleoprotein (RNP), mitochondrial antibodies, anti-Jo-1, anti-chromatin, anti-centromere B, smooth muscle actin, and antinuclear antibody (ANA), were all negative. In addition, the patient tested negative for COVID-19, influenza, and respiratory syncytial virus.

**Figure 3:**
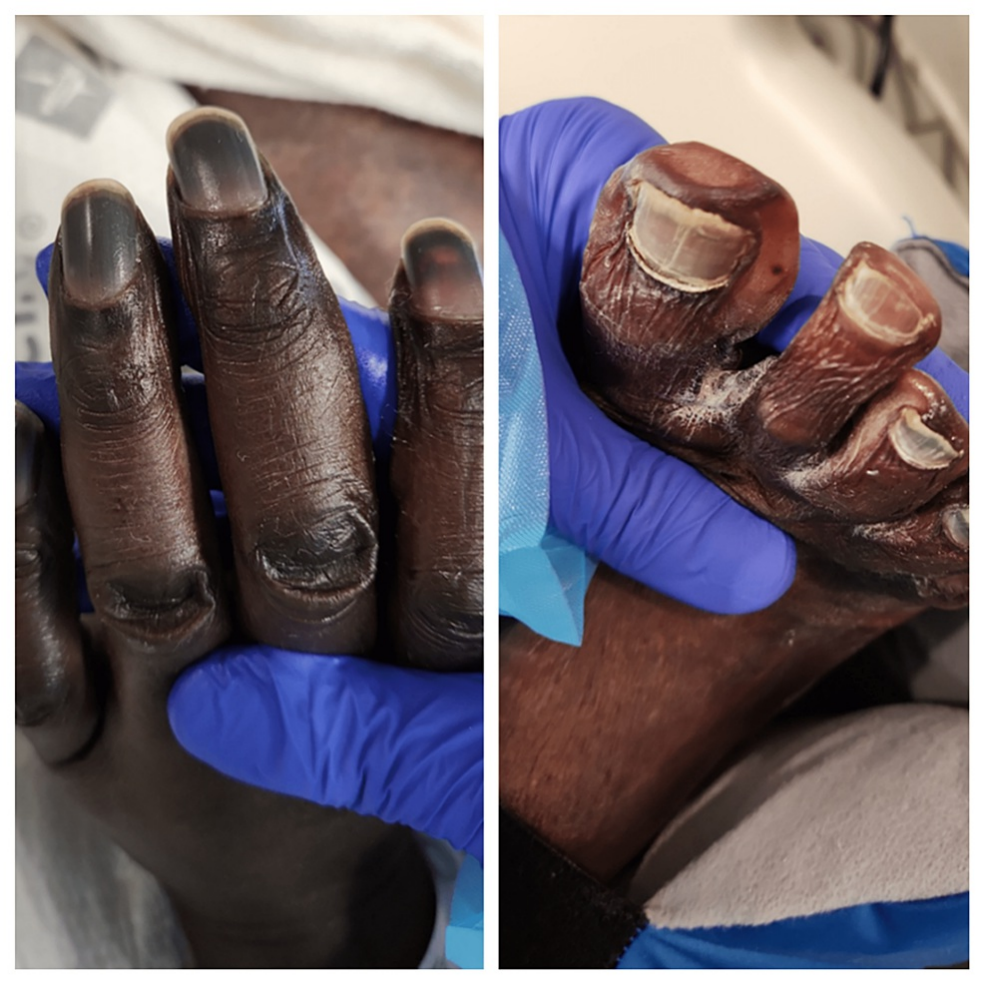
Images demonstrating digit mummification of the hands and feet

**Figure 4:**
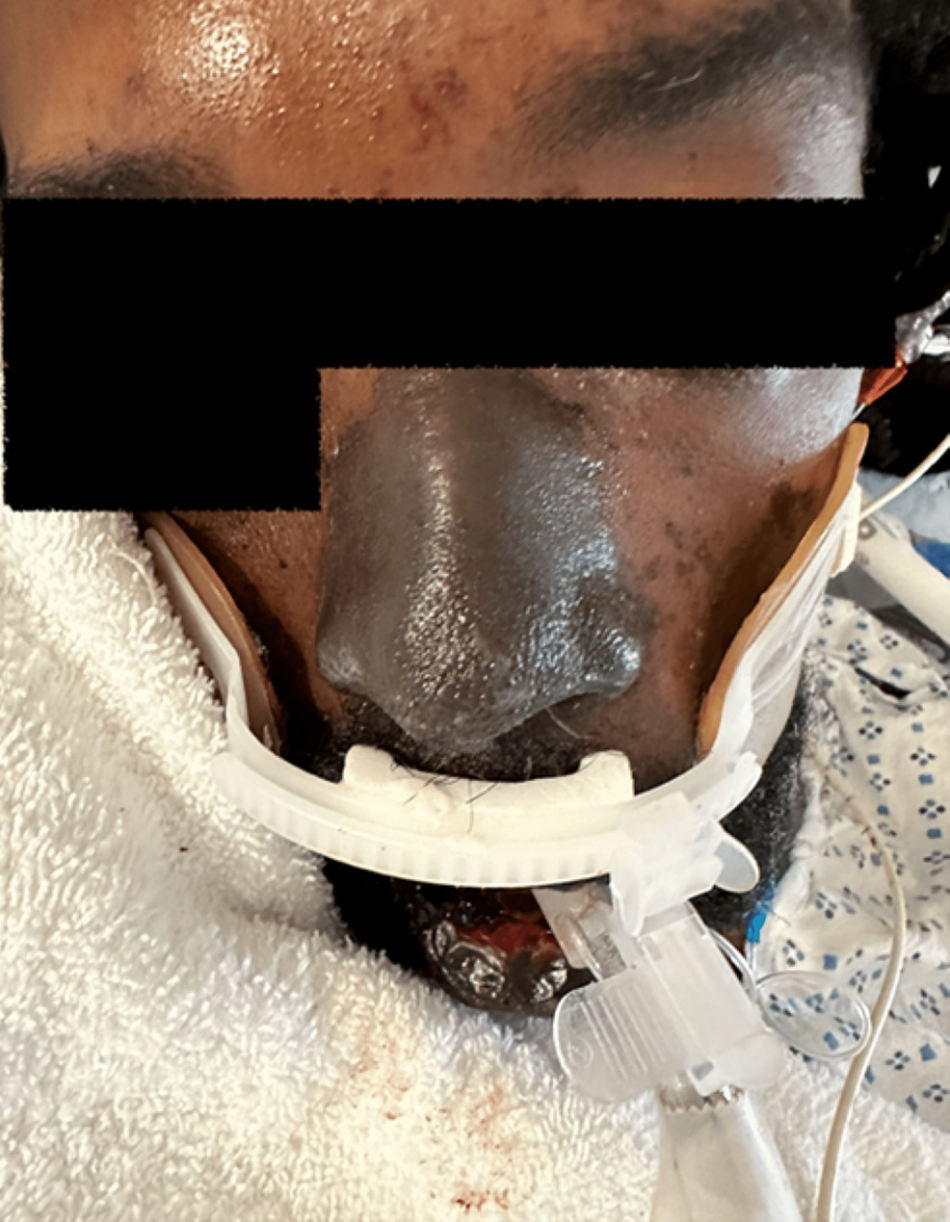
Discoloration of the lips and nose

After three days, the blood culture from admission grew *Streptococcus pneumoniae*, and the following blood cultures grew the same. All four extremities became hyperpigmented and edematous with blebs, and peripheral pulses were absent. Livedo reticularis was noted in the lower extremities, and digit mummification was pointed out on the hands and feet (Figure 3). Arterial duplex showed severe ischemia to all four extremities. Also, his lips, nose, and genitals became hyperpigmented (Figure 4). These findings were suggestive of extensive skin necrosis. The venous duplex showed a thrombus in the right posterior tibial vein. However, heparin was not started because platelets were low, ranging from 14×103/uL to 43×10^3^/uL.

The patient developed multiorgan failure with acute kidney failure with anuria for which continuous renal replacement therapy was initiated and hepatic failure for which N-acetylcysteine protocol was also started. The hepatitis panel was all negative. Cardiology evaluated the patient for elevated troponin. The cause of this was thought to be acute cardiomyopathy and non-ST elevation myocardial infarction secondary to demand ischemia and coagulopathy. Transthoracic echocardiogram demonstrated a left ventricular ejection fraction of 25%. The patient expired on day 6 from multiorgan failure secondary to septic shock in the setting of DIC.

## Discussion

PF is a life-threatening hematological emergency with rapidly progressive thrombotic sequelae, culminating in widespread hemorrhagic necrosis of the skin and DIC. Mortality is 43% [2]. It almost always involves relentless aberrant coagulation cascade activation, with either hereditary or acquired predisposition. In our case, the patient had a history of alcohol abuse predisposing him to *Streptococcus pneumoniae* bacteremia.

Alcohol impairs our body's immune response by affecting the adhesion, migration, and phagocytosis of the neutrophil-dependent killing of pneumococci. Neutrophils are the predominant phagocytic cells responsible for bacterial clearance in pneumococcal pneumonia. In rodent studies, acute alcohol exposure suppresses the expression of lung chemokines, effectively delaying the recruitment of neutrophils to the site of infection. It also causes a dose-dependent inhibition of the release of both primary and secondary granule contents. In addition, chronic alcohol use was shown to reduce both the oxidative burst and lysozymes released by polymorphonuclear leukocytes (PMNLs) [6].

Alcohol abuse has been implicated as an important risk factor for developing an invasive pneumococcal disease (IPD). A prospective study by Grau et al. [7] looked at 1,378 culture-proven cases of invasive pneumococcal disease (IPD) in adult patients from 1997 to 2011. They found that compared to the general population, patients with IPD were often alcohol abusers (21% versus 6%) [7]. In addition to being a significant risk factor in the development of invasive pneumococcal disease, alcohol abuse has been found to increase mortality by 50% in unvaccinated individuals, as demonstrated by a case-control study by Morton et al. [8] on unvaccinated veterans. Interestingly, pneumococcal serotypes 4, 11A, and 19F were more prevalent in alcohol abusers than non-abusers. Compared to the control group, alcohol abusers had less frequently received the PPV23 vaccine compared to the control group (0% versus 30%), which covers the serotypes implicated in IPD [7].

In the USA, as of 2019, there were 19,951 cases reported of IPD, a rate of 8.15 per 100,000 [9]. The CDC recommends pneumococcal vaccination for adults 19 to 64 with certain chronic medical conditions or other risk factors, including alcoholism and cigarette smoking. However, the vaccination rate in this at-risk group is 24.5% of all eligible adults [10]. Mortality from IPD depends on the severity of the disease, with 30-day mortality from sepsis estimated at 5.4% in a prospective observational study from Norway. The same study reported a mortality of 20.2% for those with severe sepsis with organ failure and 35% for patients with septic shock [11].

Pneumococcal bacteremia represents a form of invasive pneumococcal disease and is associated with high mortality, especially in immunocompromised patients and the elderly. Purpura fulminans is a rare complication and manifestation of disseminated intravascular coagulation and sepsis. It is exceedingly rare in the setting of pneumococcal bacteremia, particularly in immunocompetent individuals. Our patient's only identifiable risk factor was alcohol use. We hope that this case report can highlight the importance of pneumococcal vaccination in this population, especially in an era of vaccine hesitancy.

## Conclusions

This case report highlights the devastating consequences of alcohol abuse and its impact on the immune system, particularly when combined with an invasive bacterial infection such as *Streptococcus pneumoniae*. The patient, in this case, developed septic shock and purpura fulminans, which is a potentially life-threatening condition characterized by rapidly progressive skin necrosis and disseminated intravascular coagulation.

This case report underscores the importance of vaccination as a preventive measure against invasive pneumococcal disease, which can lead to sepsis and purpura fulminans. Vaccination against pneumococcal disease is crucial for individuals with chronic alcohol abuse and other comorbidities that can weaken the immune system. Healthcare providers should prioritize educating their patients about the importance of vaccination and ensuring that they are up-to-date on their recommended vaccinations.
